# Radiosynthesis and Preclinical Evaluation of ^18^F-Labeled Estradiol Derivatives with Different Lipophilicity for PET Imaging of Breast Cancer

**DOI:** 10.3390/cancers16152639

**Published:** 2024-07-24

**Authors:** Anna Friedel, Olaf Prante, Simone Maschauer

**Affiliations:** 1 Department of Nuclear Medicine, Molecular Imaging and Radiochemistry, Friedrich-Alexander-Universität Erlangen-Nürnberg (FAU), 91054 Erlangen, Germany; anna.friedel@fau.de (A.F.); olaf.prante@uk-erlangen.de (O.P.); 2 FAU NeW—Research Center New Bioactive Compounds, Friedrich-Alexander-Universität Erlangen-Nürnberg (FAU), 91058 Erlangen, Germany

**Keywords:** breast cancer, estrogen receptor, fluorine-18, PET imaging, ^18^F-fluoroglycosylation

## Abstract

**Simple Summary:**

Breast cancer is one of the most prevalent forms of cancer diagnosed in women worldwide. Since the estradiol receptor (ER) is overexpressed in 75% of breast tumors, it is a reasonable target for tumor diagnosis and therapy. This study focuses on the development and preclinical evaluation of readily synthesized ^18^F-labeled estradiol derivatives with different lipophilicity. The least hydrophilic derivative, ^18^F-TA-Glyco-EE, showed the highest cellular uptake in ER-positive breast cancer cells. The in vivo PET imaging of breast tumor-bearing mice demonstrated the desired rapid clearance of the tracer from the excretory organ through the liver. The in vitro autoradiography of ER-positive tumor sections confirmed the high specific binding of ^18^F-TA-Glyco-EE. In conclusion, ^18^F-TA-Glyco-EE may be a promising candidate for imaging of ER-positive breast cancer.

**Abstract:**

About 75% of breast tumors show an overexpression of the estradiol receptor (ER), making it a valuable target for tumor diagnosis and therapy. To date, 16*α*-[^18^F]fluoroestradiol (FES) is the only FDA-approved imaging probe for the positron emission tomography (PET) imaging of ER-positive (ER^+)^ breast cancer. However, FES has the drawback of a high retention in the liver. Therefore, the aim of this study was the development and preclinical evaluation of estradiol (E2) derivatives with different lipophilicity. Three ^18^F-labeled prosthetic groups (two glycosyl and one PEG azide) were chosen for conjugation with ethinyl estradiol (EE) by ^18^F-CuAAC (Cu-catalyzed azide-alkyne cycloaddition). The cellular uptake in ER^+^ MCF-7 tumor cells was highest for the less hydrophilic derivative (^18^F-TA-Glyco-EE). In nude mice bearing different breast tumors (ER^+^ MCF-7 and T47D versus ER^−^ MDA-MB-231), ^18^F-TA-Glyco-EE revealed a high uptake in the liver (13%ID/g, 30 min p.i.), which decreased over 90 min to 1.2%ID/g, indicating fast hepatobiliary clearance. The statistically significant difference of ^18^F-TA-Glyco-EE uptake in T47D compared to MDA-MB-231 tumors at 60–90 min p.i. indicated ER-specific uptake, whereas in vivo PET imaging did not provide evidence for specific uptake of ^18^F-TA-Glyco-EE in MCF-7 tumors, probably due to ER occupation by E2 after E2-dependent MCF-7 tumor growth in mice. However, in vitro autoradiography revealed a high specific binding of ^18^F-TA-Glyco-EE to ER^+^ tumor slices. We conclude that ^18^F-TA-Glyco-EE, with its increased hydrophilicity after deacetylation in the blood and thus rapid washout from non-target tissues, may be a viable alternative to FES for the PET imaging of breast cancer.

## 1. Introduction

Breast cancer is one of the most common types of cancer in the world [1]. At diagnosis, 75% of breast tumors have an overexpression of the estradiol receptor (ER), thus being classified as ER-positive (ER^+^) [2]. These tumors can respond to hormonal therapy, whereas ER-negative (ER^−^) tumors require other therapeutic methods like chemotherapy or surgery. The ER is mainly expressed as a nuclear receptor in two isoforms, ERα and ERβ, or as a membrane receptor. These subtypes show distinct tissue distribution; however, in the following, we refer to ERα as ER, since the nuclear ERα is predominantly expressed in breast cancer [3]. The steroid hormone 17*β*-estradiol (E2), as the endogenous ligand, activates the nuclear ER leading to receptor dimerization, followed by transcriptional activities and gene expression, which in turn affects the development of breast tissue [4]. The status detection of ER is routinely performed by biopsy, followed by immunohistochemistry. However, this invasive method has a low accuracy for the detection of tumor metastasis [5]. To overcome these limitations, non-invasive positron emission tomography (PET) is a viable technique to visualize the complete tumor heterogeneity by the imaging of ER^+^ lesions [5]. Various PET and single-photon emission computer tomography (SPECT) tracers labeled with, e.g., F-18 [6,7,8,9], Tc-99m [10,11] or I-131 [12,13] as a radionuclide are reported in the literature, but so far, 16*α*-[^18^F]fluoroestradiol (FES) is the only FDA-approved imaging probe for PET imaging of ER^+^ breast cancer [14]. A comprehensive review was published in 2021 that describes the long journey, starting in 1984, of the conception and design that finally led to the discovery of FES by John A. Katzenellenbogen [15].

FES uptake detected by PET correlates with ER expression, such that FES is used in clinics to predict the response of breast cancer tissue to endocrine therapy by evaluating the ER status [15]. However, FES has the drawback of having a high liver uptake and long retention in the liver, due to FES metabolism in the liver and its lipophilicity [16]. Additionally, FES and its so-far reported derivatives suffer from a fast blood clearance, resulting in low tumor uptake [17].

**Figure 1:**
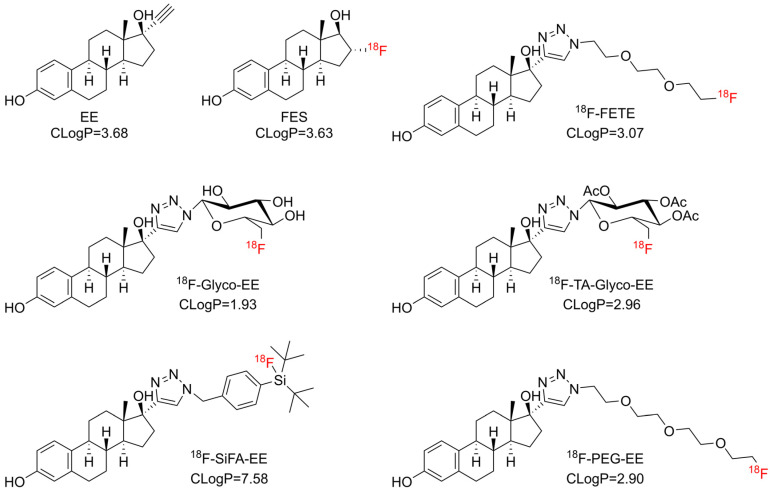
Chemical structures of FES, ^18^F-FETE [8], EE and derivatives thereof synthesized in this study aiming at different lipophilicity. CLogP values were calculated using the software ChemDraw, Version 19 (Revvity Signals Software, Revvity Inc., Waltham, MA, USA).

Derivatization with an ethinyl group in the C17 position of E2 was reported to give 17*α*-ethinylestradiol (EE), which showed a higher ER affinity compared to E2, together with improved pharmacokinetic properties due to decreased first-pass hepatic extraction [18]. Further derivatization by the introduction of a triazole ring in the C17 position by Cu-catalyzed azide-alkyne cycloaddition (CuAAC; click chemistry) resulted in a varied binding affinity for ER which largely depended on the nature of the 4-substituent of the triazole ring [19]. Xu et al. showed that the derivatization of EE by a triazole moiety maintained the ER affinity and demonstrated a specific binding to ER in the case of [^18^F]FETE, a PEGylated triazolyl derivative (Figure 1) [8], and [^125/131^I]ITE2, an E2 dimer [13].

Based on these findings, it appears that EE may be a suitable, readily commercially accessible building block for the synthesis of E2 derivatives by CuAAC with ^18^F-labeled prosthetic groups. Fluorine-18, with a convenient half-life of 109.8 min, combined with its relatively low positron energy (E_max_ = 635 keV), is the preferred radioisotope for PET imaging [20].

Therefore, the objective of the present study was to develop an alternative ^18^F-labeled positron emission tomography (PET) tracer for breast cancer imaging. The tracer was designed to be synthesized using click chemistry, exhibit sufficient ER affinity and display a favorable biodistribution with marked liver clearance. To achieve this objective, three distinct hydrophilic ^18^F-labeled prosthetic groups (two glycosyl and one PEG azide) were selected for conjugation with EE (Figure 1). Furthermore, a silicon-fluoride acceptor azide (SiFA azide) [21] was selected for comparison (Figure 1), due to its known lipophilicity and easy and fast labeling reaction by isotopic exchange of fluorine-19 [22].

## 2. Materials and Methods

### 2.1. General

All chemicals were purchased from commercial sources (ABCR GmbH (Karlsruhe, Germany), Fisher Scientific GmbH (Schwerte, Germany), Alfa Aesar (Haverhill, MA, USA), Carl Roth (Karlsruhe, Germany), Merck KGaA (Darmstadt, Germany), and Sigma Aldrich (Taufkirchen, Germany)) in the highest available quality and used without further purification. NMR spectra were acquired on a Bruker Avance Nanobay V3-I 400 MHz or a Bruker Avance III HD 600 MHz spectrometer. ESI–mass spectra were recorded using LC-MS: Thermo Scientific Dionex Ultimate 3000 UHPLC quaternary pump, autosampler and RS-diode array detector, column: Zorbax-Eclipse XDB-C8 analytical column (3.0 mm × 100 mm, 3.5 μm), flow rate 0.4 mL/min using DAD detection (230 nm; 254 nm), coupled to a Bruker Daltonics Amazon mass spectrometer. High mass accuracy and resolution experiments were performed on a Bruker Daltonics timsTOF Pro spectrometer using electrospray ionization (ESI) as an ionization source. HPLC was performed on an Agilent 1100 system (Agilent Technologies Inc., Santa Clara, CA, USA) with a quaternary pump and variable wavelength detector and radio-HPLC detector HERM LB 500 (Berthold Technologies GmbH & Co. KG, Bad Wildbad, Germany). No-carrier-added [^18^F]fluoride was produced through the ^18^O(p,n)^18^F reaction on a PETtrace 800 cyclotron (General Electric, Uppsala, Sweden) using H_2_[^18^O]O as the target at the Universitätsklinikum Würzburg (Klinik und Poliklinik für Nuklearmedizin, Radiopharmazie/PET-Zentrum, Würzburg, Germany) and transported by road to Erlangen. All radiolabeled derivatives were verified by co-injection with their respective reference compounds on the HPLC system (Figures S1–S4).

### 2.2. Syntheses

#### 2.2.1. Synthesis of Glyco-EE

17*α*-Ethinylestradiol (29.6 mg, 100 µmol, 1 eq) and 6-deoxy-6-fluoro-β-D-glucopyranosyl-1-azide [23] (Glyco-N_3_, 20.7 mg, 100 µmol, 1 eq) were dissolved in water (1.3 mL) and ethanol (500 µL). Sodium ascorbate (100 mM, 20 µL), Cu(OAc)_2_ (4 mM, 20 µL) and tris(3-hydroxypropyltriazolylmethyl)amine (THPTA, 20 mM, 20 µL) were added, and the reaction was stirred for 30 min at room temperature. The crude product was purified by HPLC (Kromasil C8, 125 × 8 mm, 20–50% acetonitrile (with 0.1% TFA) in water (with 0.1% TFA) over 20 min, 4 mL/min, t_R_ = 8.30 min), and Glyco-EE was obtained after lyophilization in a yield of 3% (1.66 mg, 3.3 µmol). Purity (HPLC): >99% (Chromolith RP18e 100 × 4.6 mm, 4 mL/min, 10–100% acetonitrile in water + 0.1% TFA, t_R_ = 2.01 min), ESI-MS: *m*/*z* 504.2 [M + H]^+^.

#### 2.2.2. Synthesis of TA-Glyco-EE

17*α*-Ethinylestradiol (21.0 mg, 70.8 µmol, 1 eq) and 2,3,4-tri-*O*-acetyl-6-deoxy-6-fluoro-β-D-glucopyranosyl-1-azide [23] (TA-Glyco-N_3_, 23.6 mg, 70.8 µmol, 1 eq) were dissolved in DMSO (500 µL). A solution of Cu(II) sulfate (0.2 M, 40 µL) and sodium ascorbate (0.6 M, 40 µL) was added, and the reaction was stirred at 40 °C overnight. The crude product was prepurified by column chromatography (Silica gel 60, n-hexane/ethyl acetate = 3/2), followed by purification with flash column chromatography (Büchi Ecoflex C18, 50 µm, spherical, 20 g, 25–50% acetonitrile in water + 0.1% TFA). After lyophilization of the product fraction, TA-Glyco-EE was obtained as a white solid in a yield of 65% (29.2 mg, 46.4 µmol). Purity (HPLC): >99% (Chromolith RP18e 100 × 4.6 mm, 4 mL/min, 10–100% acetonitrile in water + 0.1% TFA, t_R_ = 2.69 min), HR-MS *m*/*z* calculated for C_32_H_40_FN_3_O_9_ 630.2821 [M + H]^+^, found 630.2827 [M + H]^+^. ^1^H NMR (600 MHz, DMSO-d_6_) δ 8.94 (s, 1H), 8.11 (s, 1H), 6.94 (d, J = 8.4 Hz, 1H), 6.46 (dd, J = 8.4, 2.6 Hz, 1H), 6.41 (d, J = 2.6 Hz, 1H), 6.35 (d, J = 9.3 Hz, 1H), 5.68 (t, J = 9.5 Hz, 1H), 5.56 (t, J = 9.5 Hz, 1H), 5.21 (t, J = 9.8 Hz, 1H), 4.62–4.58 (m, 0.5 H), 4.55–4.49 (m, 1H), 4.45–4.40 (m, 1H), 4.39–4.36 (m, 0.5 H), 2.75–2.64 (m, 2H), 2.43–2.37 (m, 1H), 2.05 (s, 3H), 1.97 (s, 3H), 1.92 (ddd, J = 13.3, 11.7, 3.8 Hz, 1H), 1.86–1.78 (m, 2H), 1.74 (s, 3H), 1.73–1.70 (m, 1H), 1.53–1.47 (m, 1H), 1.46–1.38 (m, 2H), 1.35–1.14 (m, 4H), 0.91 (s, 3H), 0.43 (td, J = 13.0, 4.2 Hz, 1H). ^13^C NMR (151 MHz, DMSO-d_6_) δ 169.5, 169.3, 168.2, 154.8, 154.8, 137.1, 130.3, 125.9, 121.3, 114.9, 112.6, 83.7, 81.2 (d, J = 119.4 Hz), 80.5, 73.9 (d, J = 18.2 Hz), 72.2, 70.1, 66.7 (d, J = 7.2 Hz), 47.5, 46.6, 43.2, 39.2, 36.8, 32.5, 29.2, 27.2, 26.1, 23.6, 20.4, 20.2, 19.8, 14.2.

#### 2.2.3. Synthesis of PEG-EE

17*α*-Ethinylestradiol (35.2 mg, 118.8 µmol, 1 eq) and 1-azido-2-(2-(2-(2-fluoroethoxy)ethoxy)ethoxy)ethan [24] (PEG-N_3_, 26.9 mg, 121.4 µmol, 1.02 eq) were dissolved in DMSO (700 µL). A solution of Cu(II) sulfate (0.2 M, 80 µL) and sodium ascorbate (0.6 M, 80 µL) was added, and the reaction was stirred at 45 °C for 5.5 h. The crude product was purified by flash column chromatography (Büchi Ecoflex C18, 50 µm, spherical, 40 g; 15–50% acetonitrile in water + 0.1% TFA). After lyophilization, PEG-EE was obtained as a yellow-brown solid in a yield of 89% (54.8 mg, 105.9 µmol). Purity (HPLC): >99% (Chromolith RP18e 100 × 4.6 mm, 4 mL/min, 10–100% acetonitrile in water + 0.1% TFA, t_R_ = 2.24 min), HR-MS *m*/*z* calculated for C_28_H_40_FN_3_O_5_ 518.3025 [M + H]^+^, found 518.3029 [M + H]^+^. ^1^H NMR (600 MHz, DMSO-d_6_) δ 8.94 (br s, 1H), 7.82 (s, 1H), 6.95 (d, J = 8.6 Hz, 1H), 6.46 (dd, J = 8.4, 2.6 Hz, 1H), 6.41 (d, J = 2.6 Hz, 1H), 4.53 (t, J = 4.0 Hz, 1H), 4.49 (t, J = 5.3 Hz, 2H), 4.45 (t, J = 4.0 Hz, 1H), 3.82 (t, J = 5.3 Hz, 2H), 3.64 (t, J = 4.1 Hz, 1H), 3.59 (t, J = 4.1 Hz, 1H), 3.52 (dt, J = 6.3, 2.6 Hz, 4H), 3.51–3.47 (m, 4H), 2.77–2.64 (m, 2H), 2.40–2.31 (m, 1H), 2.11–2.04 (m, 1H), 1.93 (ddd, J = 13.3, 11.8, 3.7 Hz, 1H), 1.87–1.71 (m, 3H), 1.61 (td, J = 11.4, 7.5 Hz, 1H), 1.50–1.37 (m, 2H), 1.33 (qd, J = 11.0, 2.6 Hz, 1H), 1.29–1.16 (m, 2H), 0.92 (s, 3H), 0.59 (td, J = 13.0, 4.2 Hz, 1H). ^13^C NMR (151 MHz, DMSO-d_6_) δ 154.8, 153.9, 137.1, 130.4, 125.9, 123.0, 114.9, 112.6, 83.0 (d, J = 165.6 Hz), 81.0, 69.8 (2C), 69.7, 69.7, 69.6, 68.9, 49.2, 47.5, 46.7, 43.2, 39.3, 37.1, 32.6, 29.2, 27.2, 26.1, 23.5, 14.4.

#### 2.2.4. Synthesis of SiFA-EE

17*α*-Ethinylestradiol (25.0 mg, 84.3 µmol, 1 eq) and 4-(di-*tert*-butylfluorosilyl)benzylazide [21] (SiFA azide, 25.2 mg, 85.9 µmol, 1.02 eq) were dissolved in DMSO (440 µL). A solution of Cu(II) sulfate (0.2 M, 80 µL) and sodium ascorbate (0.6 M, 80 µL) was added, and the reaction was stirred at 45 °C for 2 d. The crude product was purified by flash column chromatography (Büchi Select C18, 30 µm, spherical, 4 g; 55–70% acetonitrile in water + 0.1% TFA). After lyophilization, SiFA-EE was obtained as a white solid in a yield of 46% (22.7 mg, 38.6 µmol). Purity (HPLC): 97%, (Chromolith RP18e 100 × 4.6 mm, 4 mL/min, 10–100% acetonitrile in water + 0.1% TFA, t_R_ = 3.91 min) HR-MS *m*/*z* calculated for C_35_H_48_FN_3_O_2_Si 590.3573 [M + H]^+^, found 590.3576 [M + H]^+^. ^1^H NMR (600 MHz, CDCl_3_) δ 7.61 (d, J = 8.0 Hz, 2H), 7.39 (s, 1H), 7.26 (s, overlap with CHCl_3_), 7.25 (s, 1H), 7.05 (dd, J = 8.5, 1.0 Hz, 1H), 6.59 (dd, J = 8.5, 2.8 Hz, 1H), 6.54 (d, J = 2.7 Hz, 1H), 5.56 (s, 2H), 2.88–2.71 (m, 3H), 2.40–2.31 (m, 1H), 2.15–2.11 (m, 2H), 1.92–1.87 (m, 3H), 1.68–1.61 (m, 1H), 1.58–1.36 (m, 5H), 1.32–1.24 (m, 2H), 1.03 (dd, J = 5.5, 1.2 Hz, 21H).

### 2.3. Radiosyntheses

#### 2.3.1. Radiosynthesis of ^18^F-TA-Glyco-EE and ^18^F-Glyco-EE

The prosthetic group 2,3,4-tri-*O*-acetyl-6-deoxy-6-[^18^F]fluoroglucopyranosyl azide (TA-Glyco-N_3_) was synthesized, purified by HPLC and isolated using a C18 cartridge (SepPak light, Waters GmbH, Eschborn, Germany) as described previously [23]. TA-Glyco-N_3_ was eluted from the cartridge with 600 µL ethanol into an Eppendorf vial, containing CuSO_4_ (0.2 M, 20 µL), sodium ascorbate (0.6 M, 20 µL) and 17*α*-ethinylestradiol (10 mM, 40 µL). The reaction mixture was heated at 60 °C for 10 min, and the solvent was continuously reduced under a stream of helium. For the radiosynthesis of ^18^F-TA-Glyco-EE, water (600 µL, 0.1% TFA) and acetonitrile (100 µL) were added to adjust the reaction volume to 1 mL, and the product was subsequently purified by semi-preparative HPLC (Chromolith RP-18e, 100 × 10 mm, 30–75% acetonitrile (with 0.1% TFA) in water (with 0.1% TFA) over 30 min, 4 mL/min, t_R_ = 11.11 min). The product fraction containing ^18^F-TA-Glyco-EE was diluted with water (16 mL) and passed through a preconditioned (acetonitrile followed by water, each 5 mL) SepPak light C18 cartridge (Waters GmbH, Eschborn, Germany). ^18^F-TA-Glyco-EE was eluted from the cartridge with ethanol (1 mL). and was obtained in an activity yield (AY) of 25% (referred to [^18^F]fluoride) after 77 min and identified by co-injection with TA-Glyco-EE by HPLC.

For the radiosynthesis of ^18^F-Glyco-EE, ^18^F-TA-Glyco-EE was deacetylated after the click reaction described above by the addition of NaOH (0.5 M, 100 µL) for 5 min at 60 °C. Afterwards, the reaction mixture was neutralized with HCl (0.5 M, 100 µL), the reaction volume was adjusted to 1 mL with water, and the ^18^F-Glyco-EE was purified by semi-preparative HPLC (Kromasil C8, 125 × 8 mm, 20–50% acetonitrile (with 0.1% TFA) in water (with 0.1% TFA) over 20 min, 4 mL/min, t_R_ = 8.40 min). The product fraction was diluted with water (16 mL) and passed through a preconditioned (acetonitrile followed by water, each 5 mL) SepPak light C18 cartridge (Waters GmbH, Eschborn, Germany). The cartridge was eluted with ethanol (1 mL). ^18^F-Glyco-EE was obtained in an AY of 22% (referred to [^18^F]fluoride) after 90 min and identified by co-injection with Glyco-EE by HPLC.

#### 2.3.2. Radiosynthesis of ^18^F-PEG-EE

[^18^F]Fluoride was trapped on a QMA cartridge (Waters GmbH, Eschborn, Germany) and eluted with a solution of Kryptofix 2.2.2 (15 mg) and K_2_CO_3_ (1 M, 15 µL) in acetonitrile (900 µL) and water (100 µL) into a reaction vial. The solvent was evaporated under a stream of helium at 85 °C and azeotropically dried with anhydrous acetonitrile (3× 500 µL). The labeling precursor 2-(2-(2-(2-azidoethoxy)ethoxy)ethoxy)ethyl methanesulfonate [24] (8 mg, pre-dried under vacuum overnight) in acetonitrile (400 µL) was added, and the reaction was stirred at 85 °C for 5 min. By adding water (600 µL, 0.1% TFA), the reaction volume was adjusted to 1 mL, and the reaction solution was subjected to semi-preparative HPLC (Kromasil C8, 125 × 10 mm, 10–55% acetonitrile (with 0.1% TFA) in water (with 0.1% TFA) over 20 min, 4 mL/min, t_R_ = 7.07 min). The product fraction containing 1-azido-2-(2-(2-(2-[^18^F]fluoroethoxy)ethoxy)ethoxy)ethan (PEG-N_3_) was diluted with water (16 mL) and passed through a preconditioned (acetonitrile followed by water, each 5 mL) StrataX C_18_ (60 mg) cartridge (Phenomenex, Torrance, CA, USA). The cartridge was eluted with 600 µL DMSO into a reaction vial, containing CuSO_4_ (0.2 M, 20 µL), sodium ascorbate (0.6 M, 20 µL) and 17*α*-ethinylestradiol (10 mM, 40 µL). The reaction mixture was heated in a heating block for 15 min at 60 °C. By adding water (400 µL, 0.1% TFA), the reaction volume was adjusted to 1 mL, and ^18^F-PEG-EE was subsequently purified by semi-preparative HPLC (Kromasil C8, 125 × 10 mm, 30–75% acetonitrile (with 0.1% TFA) in water (with 0.1% TFA) over 30 min, 4 mL/min, t_R_ = 9.5 min). The product fraction was diluted with water (16 mL) and passed through a preconditioned (acetonitrile followed by water, each 5 mL) SepPak light C18 cartridge (Waters GmbH, Eschborn, Germany). ^18^F-PEG-EE was eluted from the cartridge with ethanol (1 mL) and was obtained in an AY of 19% (referred to [^18^F]fluoride) after 81 min and identified by co-injection with PEG-EE by HPLC.

#### 2.3.3. Radiosynthesis of ^18^F-SiFA-EE

[^18^F]Fluoride was trapped on a QMA cartridge and eluted with a solution of Kryptofix 2.2.2 (10 mg), K_2_CO_3_ (0.1 M, 17.5 µL) and KH_2_PO_4_ (0.1 M, 17.5 µL) in acetonitrile (800 µL) and water (165 µL) into a reaction vial. The solvent was evaporated under a stream of helium at 85 °C and azeotropically dried with anhydrous acetonitrile (3 × 500 µL). The labeling precursor SiFA-EE [21] (10 nmol) was added to 300 µL dry acetonitrile, and the reaction was stirred for 5 min at room temperature. The reaction was quenched with NaH_2_PO_4_ (0.1 M, pH = 4–5, 10 mL) and passed through a preconditioned (acetonitrile followed by water, each 5 mL) SepPak light C18 cartridge (Waters GmbH, Eschborn, Germany). The cartridge was washed with water (5 mL), ^18^F-SiFA-EE was eluted from the cartridge with ethanol (1 mL), and the solvent was evaporated under reduced pressure. The product was redissolved in Hanks′ Balanced Salt Solution (HBSS) (pH = 6.4) for further use. ^18^F-SiFA-EE was obtained in an AY of 44% after 27 min and identified by co-injection with SiFA-EE by HPLC.

### 2.4. In Vitro Studies

#### 2.4.1. Determination of logD_7.4_

For the determination of the octanol–water coefficient, an aliquot of the radiotracer was added to PBS (500 µL, pH 7.4) and n-octanol (500 µL) in an Eppendorf tube. The mixture was vortexed and subsequently centrifuged, each for 1 min. Three samples (each 100 µL) were taken from both the PBS and the n-octanol layer and measured using a γ-counter (Wallac Wizard, PerkinElmer, Waltham, MA, USA). The logD_7.4_ was expressed as log(cpm_octanol_/cpm_PBS_) and reported as mean ± SD. The determination of logD_7.4_ was performed in three independent experiments, each performed in triplicate.

#### 2.4.2. Stability in Serum and Plasma In Vitro

An aliquot of the formulated tracer solution (40 µL containing approx. 10 MBq in 0.9% NaCl) was incubated with 200 µL of human plasma (Hölzel Diagnostika Handels GmbH, from CosmoBio USA, Carlsbad, CA, USA) or human serum (Biochrom GmbH, Merck KGaA, Darmstadt, Germany), respectively, at 37 °C. After various time points (5–120 min), aliquots (30 µL) were taken and quenched in methanol or 10% TFA (30 µL). The samples were centrifuged for 3 min, and subsequently the supernatants were analyzed by radio-HPLC (Chromolith RP-18e, 100 × 4.6 mm, 10–100% acetonitrile (with 0.1% TFA) in water (with 0.1% TFA) over 5 min, 4 mL/min).

#### 2.4.3. Plasma Protein Binding

MicroSPin G-50 columns (Cytiva, Merck KGaA, Darmstadt, Germany) were preconditioned according to the manufacturer’s protocol and the experiment was performed as follows. In brief, an aliquot of the tracer (1 µL, approx. 250 kBq) was added to NaCl (0.9%, 200 µL, negative control) or human plasma (200 µL) and incubated for 10 min at 37 °C. A sample from the plasma or negative control (40 µL) was carefully added to the resin, and the column was placed in an Eppendorf tube. After centrifugation (2 min, 2000× *g*), the eluate and the resin were collected separately. Both were measured for radioactivity using a *γ*-counter (Wallac Wizard, PerkinElmer, Waltham, MA, USA), and the radioactivity eluted from the resin was calculated as a percentage of the total amount of radioactivity. The determination of plasma protein binding was performed twice in duplicate.

#### 2.4.4. Cell Lines

The human breast cancer cell lines MCF-7 and T47D were used as positive controls (ER^+^), whereas the human breast cancer cell line MDA-MB-231 was used as a negative control (ER^−^) [25]. MCF-7 cells were obtained from DSMZ (Leibniz Institute, DSMZ—German Collection of Microorganisms and Cell Cultures GmbH, Braunschweig, Germany); MDA-MB-231 and T47D cells were obtained from the American Type Culture Collection (ATCC, Manassas, VA, USA). MCF-7 cells were cultured in DMEM/Ham F-12 containing 10% FBS and 1% *L*-glutamine. MDA-MB-231 cells were cultured in DMEM containing 10% FBS and 1% *L*-glutamine, and T47D cells were cultured in RPMI 1640 containing 10% FBS and 1% *L*-glutamine. All cells were cultured under sterile conditions in a humidified atmosphere containing 5% CO_2_ at 37 °C. Cells were routinely passaged twice a week.

#### 2.4.5. Stability in Cells

^18^F-TA-Glyco-EE was incubated for 90 min at 37 °C with MCF-7 cells (350,000 cells/well, seeded one day prior to the experiment in 24-well plates) in cell culture medium. After washing twice with ice-cold PBS, the cells were scraped off, and 200 µL PBS was added for resuspension. The cell suspension was transferred to Eppendorf tubes, homogenized using ultrasound (Bandelin sonoplus HD2070 ultrasonic homogenizer, room temperature, BANDELIN electronic GmbH, Berlin, Germany) and then centrifuged. The supernatant was quenched in 10% TFA, and after centrifugation the resulting supernatant was analyzed by radio-HPLC (Chromolith RP-18e, 100 × 4.6 mm, 10–100% acetonitrile (containing 0.1% TFA) in water (containing 0.1% TFA) over 5 min, 4 mL/min).

#### 2.4.6. Cellular Uptake Assays

One day prior to the experiment, MCF-7 and MDA-MB-231 cells were seeded in 24-well plates (350,000 cells/well). The cells were washed once with cell medium. The cells were incubated with the respective radiotracer in cell medium (510 µL) for 10, 20, 30, 60, 90, 120 min at 37 °C in the absence and presence of estradiol (E2, final concentration of 72 µM). After incubation, the plates were set on ice and washed with ice-cold PBS twice, following cell lysis and detachment with NaOH (0.1 M, 750 µL, 37 °C). The cells were transferred to counting tubes, and the radioactivity was measured using a *γ*-counter (Wallac Wizard, PerkinElmer, Waltham, MA, USA). The protein concentration of each well was determined in duplicate using Bradford reagent (Bio-Rad Laboratories GmbH, Feldkirchen, Germany). The cellular uptake assay was performed in 2–4 independent experiments, each performed in quadruplicate for each time point.

#### 2.4.7. Competitive Cellular Uptake

Competitive uptake experiments were performed using ^18^F-TA-Glyco-EE as a radioligand. One day prior to the experiment, MCF-7 cells were seeded in 96-well plates (50,000 cells/well). The cell medium was removed, and the respective estradiol derivative, as a competitor of the radioligand, was added in concentrations from 0 to 500 µM in 90 µL cell culture medium each. After ^18^F-TA Glyco-EE (10 µL, approx. 1 MBq/well) was added, the incubation proceeded for 60 min at 37 °C. The plate was set on ice and washed with ice-cold PBS, followed by cell lysis and detachment with NaOH (0.1 M, 200 µL, 37 °C). The cells were transferred into counting tubes, and the radioactivity was measured using a *γ*-counter (Wallac Wizard, PerkinElmer, Waltham, MA, USA). One single experiment was performed in triplicate.

### 2.5. In Vivo Studies

#### 2.5.1. Animal Models

All mouse experiments were approved by the local animal protection authorities (Government of Lower Franconia, Germany, No. 55.2 2532-2-279) and performed at the FAU in accordance with the relevant E.U. guidelines and regulations. 

Female NMRI nude mice (Crl:NMRI-Foxn1^nu^) were purchased from Charles River (Charles River Laboratories Inc., Wilmington, MA, USA) at the age of four weeks and kept in groups of four to five animals in individually ventilated cages in a twelve hours dark/light cycle, with unlimited access to water and standard chow. At the age of nine to ten weeks, one 17β-estradiol pellet per animal (0.72 mg per pellet, 3 mm diameter) with a 60-day release time (Innovative Research of America, Sarasota, FL, USA) was subcutaneously implanted on the back under isoflurane anesthesia. After three days, approximately 2–3 × 10^6^ MCF-7, T47D or MDA-MB-231 tumor cells (in 50 µL PBS) were mixed with Matrigel (50 µL, BD Biosciences, Becton, Dickinson and Company, Franklin Lakes, NJ, USA) and injected subcutaneously to the back of the mice. Tumor diameters and weight of the animals were recorded five times a week. Biodistribution or imaging studies were performed three–four weeks after the inoculation of the cells.

#### 2.5.2. Biodistribution

For biodistribution experiments, nude mice bearing T47D and MDA-MB-231 tumors (*n* = 3, per group) were injected with ^18^F-TA-Glyco-EE (2–4 MBq) under isoflurane anesthesia. After 30, 60 or 90 min, the mice were sacrificed by cervical dislocation. Blood, as well as organs and tissue from the lung, liver, kidneys, heart, spleen, brain, muscle, femur and tumors, were harvested and measured for radioactivity in the γ-counter (Wallac Wizard, PerkinElmer, Waltham, MA, USA). The samples were weighed, and the radioactivity of each tissue sample was calculated as a percentage of the total injected dose per gram tissue (%ID/g).

#### 2.5.3. PET Imaging

Small animal PET scans were performed on an Inveon™ microPET scanner (Siemens Healthineers AG, Erlangen, Germany) under isoflurane anesthesia (3%) using four MCF-7 tumor-bearing mice. ^18^F-TA-Glyco-EE (2–4 MBq in about 100 µL of saline) was intravenously injected into the tail vein under isoflurane anesthesia (3–4%). Dynamic images were acquired for 60 min, starting with the injection of the radiotracer. For displacement studies, the same mice were injected with fulvestrant (0.5 mg, s.c.) directly after the first PET scan, and two days later, ^18^F-TA-Glyco-EE (2–4 MBq in about 100 µL of saline) was injected, and a second dynamic PET scan was performed. Images were corrected for decay and attenuation, and MAP (iterative maximum a posteriori) images were reconstructed (12 × 10 s, 3 × 1 min, 5 × 5 min, 3 × 10 min, a total of 23 frames) using the built-in software of the PET scanner. An evaluation of the MAP images was conducted using the software Pmod (Version 3.6, PMOD Technologies LLC, Fällanden, Switzerland, Part of Bruker’s Preclinical Imaging Division). Regions of interest (ROIs) were drawn over the tumor, and the mean radioactivity concentration within these regions was obtained from the mean values and calculated as a percentage of the total injected dose per gram tissue (%ID/g).

### 2.6. In Vitro Autoradiography

MCF-7, MDA-MB231 and T47D tumors grown in nude mice were collected and frozen in a hexane/dry ice bath (−70 °C). Tumor slices (14 µm) were prepared on a cryostat microtome HM 500 O (Microm, Walldorf, Germany), thaw-mounted on HistoBond^®^ adhesive glass slides (Marienfeld, Lauda-Königshofen, Germany) and stored at −80 °C. For in vitro autoradiography studies, the tumor tissue sections were thawed and pre-incubated in incubation buffer (50 mM Tris HCl, 5 mM MgCl_2_, 0.2% bovine serum albumin (BSA), pH 7.4) at room temperature for 15 min. Then, the sections were transferred to incubation buffer containing ^18^F-TA-Glyco-EE (3–5 MBq) in a total volume of 70 mL. For the determination of non-specific binding, separate sections were incubated with ^18^F-TA-Glyco-EE in the presence of E2 (72 µM). After an incubation time of 60 min, the sections were washed three times with incubation buffer, followed by a short dip in ice-cold distilled water. The slices were carefully dried in a stream of warm air and finally placed on a phosphor imager screen (Fuji Imaging Plate BAS-IP SR 2025 E) overnight prior to readout (30 µm resolution) using the HD-CR-35 Bio scanner (Elysia-Raytest, Straubenhardt, Germany). The image analysis was performed using the software AIDA (version 4.21, Elysia-Raytest, Straubenhardt, Germany). Regions of interest were analyzed from 3–4 tumor slices from the MCF-7, MDA-MB231 and T47D tumors, and mean values of the intensity per area were calculated.

## 3. Results

### 3.1. Chemistry and Radiochemistry

**Figure 2:**
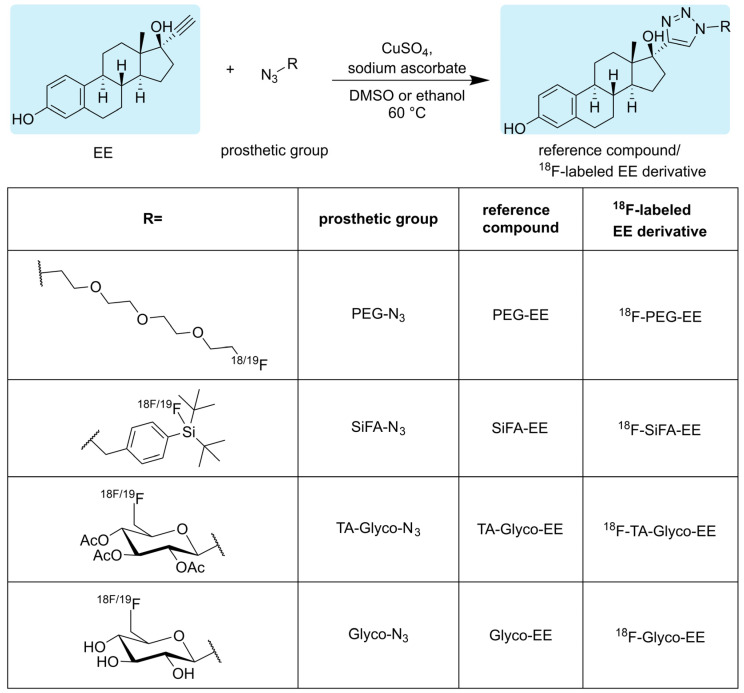
Click chemistry-based synthesis of triazolyl derivatives of estradiol with different fluorine-containing substituents (R) in the 4-position of the triazole ring and the respective radiosynthesis of their ^18^F-labeled analogs using ^18^F-labeled azides as prosthetic groups.

The reference compounds SiFA-EE, PEG-EE, TA-Glyco-EE and Glyco-EE were obtained, applying a straightforward CuAAC using commercially available EE together with the respective azides (Figure 2). The CuAAC was performed in DMSO or ethanol as solvents in the presence of CuSO_4_ as a catalyst and sodium ascorbate as a reducing agent. All compounds were purified by semi-preparative HPLC or flash column chromatography and obtained in a high chemical purity of at least 97%, as determined by HPLC. 

Applying a similar straightforward approach to the radiosynthesis of the series of ^18^F-labeled triazolyl conjugated analogs of estradiol (Figure 2; ^18^F-TA-Glyco-EE, ^18^F-Glyco-EE, ^18^F-PEG-EE and ^18^F-SiFA-EE), we envisaged the use of small ^18^F-labeled azide-bearing prosthetic groups that could be reliably conjugated to the readily available alkyne derivative of estradiol (EE) under mild click chemistry reaction conditions. All ^18^F-labeled azides used in this study as prosthetic groups had been previously described in sufficient detail in the literature [23,24,26], and their ^18^F-syntheses were successfully established in our laboratory: The glycosyl prosthetic group 2,3,4-tri-*O*-acetyl-6-deoxy-6-[^18^F]fluoroglucopyranosyl azide (TA-Glyco-N_3_) was developed in our laboratory by Maschauer et al. [23], and applied to the radiosynthesis of ^18^F-TA-Glyco-EE and ^18^F-Glyco-EE by CuAAC in ethanol using 400 nmol of EE. After isolation by semi-preparative HPLC, ^18^F-TA-Glyco-EE was obtained in an AY of 25% (referred to [^18^F]fluoride) after a total synthesis time of 77 min, with an A_m_ of up to 57 GBq/µmol. The deacetylation of ^18^F-TA-Glyco-EE, followed by semi-preparative HPLC isolation, led to ^18^F-Glyco-EE in an AY of 22% (referred to [^18^F]fluoride) after a total synthesis time of 90 min.

The ^18^F-synthesis of the alternative hydrophilic ^18^F-labeled PEG-N_3_ was achieved starting from the precursor 2-(2-(2-(2-azidoethoxy)ethoxy)ethoxy)ethyl methanesulfonate [24]. After ^18^F-fluorination, the ^18^F-labeled PEG-N_3_ was conjugated to EE by CuAAC in DMSO. ^18^F-PEG-EE was purified by semi-preparative HPLC and obtained in an AY of 19% (referred to [^18^F]fluoride) after a total synthesis time of 81 min, with an A_m_ of up to 18 GBq/µmol.

Aiming at a more lipophilic derivatization of EE for comparison to the above-described hydrophilic derivatization, the well-known isotopic ^19^F-^18^F exchange reaction according to Schirrmacher et al. [22] was applied to the radiosynthesis of ^18^F-SiFA-EE, starting from ^19^F-SiFA-EE. After cartridge purification and reformulation in HBSS (pH 6.4), ^18^F-SiFA-EE was obtained in an AY of 44% (referred to [^18^F]fluoride) after a total synthesis time of 27 min, with an A_m_ of up to 28 GBq/µmol. It should be noted here that the obtained A_m_ of ^18^F-SiFA-EE was strongly dependent on the starting activity. In addition, it was found that ^18^F-SiFA-EE was highly amenable to hydrolysis of the Si-^18^F bond even under slightly acidic conditions.

### 3.2. In Vitro Characterization

**Figure 3:**
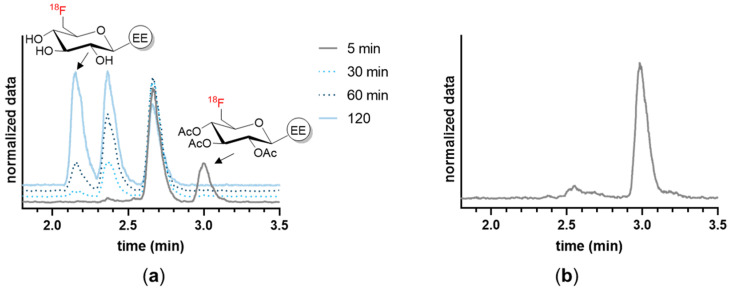
HPLC analysis of the stability of ^18^F-TA-Glyco-EE. (**a**) The stability of ^18^F-TA-Glyco-EE in human serum was determined after various incubation periods, indicating time-dependent cleavage of the acetyl groups. (**b**) The incubation of MCF-7 cells with ^18^F-TA-Glyco-EE for 90 min at 37 °C confirmed that intact ^18^F-TA-Glyco-EE was intracellularly detected, without significant deacetylation in the absence of serum.

The ^18^F-labeled EE derivatives were compared in terms of their in vitro properties as shown in Table 1. ^18^F-SiFA-EE showed high instability in both human serum and plasma (<50% after 10 min), and had therefore to be excluded from further in vitro evaluation. The determination of the octanol–buffer coefficient (logD_7.4_) confirmed the attempt to provide EE derivatives with increased hydrophilicity. Compared to EE (calculated logK_ow_ = 3.7) [27], the ^18^F-labeled EE derivatives showed lower logD_7.4_ values, due to the conjugation of hydrophilic prosthetic groups in descending order: ^18^F-TA-Glyco-EE > ^18^F-PEG-EE > ^18^F-Glyco-EE (Table 1). Both compounds, ^18^F-PEG-EE and ^18^F-TA-Glyco-EE, showed high plasma protein binding of 71% and 79%, respectively. The in vitro stability of the EE derivatives in human serum and plasma at 37 °C was analyzed by radio-HPLC. ^18^F-PEG-EE and ^18^F-Glyco-EE were stable in both plasma and serum over 120 min. As expected, triacetylated ^18^F-TA-Glyco-EE was rapidly deacetylated in serum and plasma, leading to the formation of ^18^F-Glyco-EE and partially deacetylated intermediates over 120 min (Figure 3a). In addition, the 90 min incubation of MCF-7 cells with ^18^F-TA-Glyco-EE and the HPLC analysis of the intracellular radioactivity confirmed that intact ^18^F-TA-Glyco-EE accumulated in the cells, without any significant deacetylation in the absence of serum (Figure 3b).

**Figure 4:**
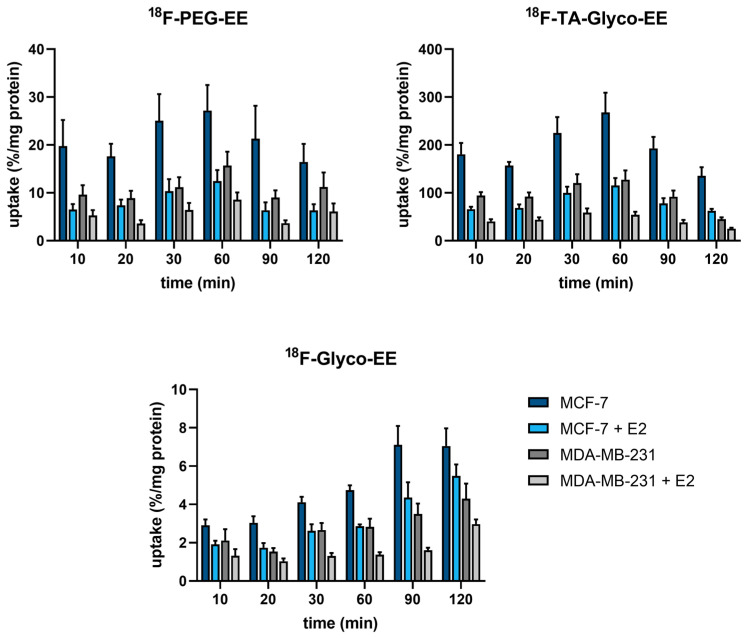
Cellular uptake (in %/mg) of the different ^18^F-labeled EE derivatives in ER^+^ MCF-7 cells and ER^−^ MDA-MB-231 cells in the absence and presence of E2 (72 µM). Each bar represents the mean ± standard error of the mean (SEM) of 2–4 independent experiments, each performed in quadruplicate.

The ^18^F-labeled EE derivatives ^18^F-TA-Glyco-EE, ^18^F-Glyco-EE, and ^18^F-PEG-EE, with favorable in vitro properties and sufficient stability, were further evaluated in cellular uptake experiments. The human breast adenocarcinoma cell line MCF-7 was used as the ER^+^ control, whereas the human breast carcinoma cell line MDA-MB-231 was used as the ER^−^ control [25]. The cells were incubated for a maximum of 120 min with the respective radiotracer in the absence or presence of E2 (72 µM) as a blocking reagent to determine non-specific (ER-independent) tracer uptake (Figure 4). All radioligands exhibited specific ER-dependent uptake in ER^+^ cells at 60–90 min of incubation that was blocked in the presence of E2 to the level of ER^−^ cells. However, a relatively high amount of non-specific uptake in ER^−^ cells was observed that could be further blocked by the addition of E2. Furthermore, the radiotracers clearly showed a time-dependent total uptake in both ER^+^ and ER^−^ cells. The most hydrophilic compound, ^18^F-Glyco-EE, showed a minimal total uptake steadily ascending over time to reach 7%/mg after 90 min, as well as the lowest specific uptake compared to ^18^F-PEG-EE and ^18^F-TA-Glyco-EE. ^18^F-PEG-EE, on the other hand, showed a four times higher total uptake (27%/mg after 60 min), in addition to a higher specific uptake compared to ^18^F-Glyco-EE. In comparison to ^18^F-PEG-EE and ^18^F-Glyco-EE, ^18^F-TA-Glyco-EE showed a 10-fold higher uptake and thus the highest specific uptake in ER^+^ cells (270%/mg after 60 min). Based on these results, ^18^F-TA-Glyco-EE was used as a radioligand for further in vitro and in vivo studies.

**Figure 5:**
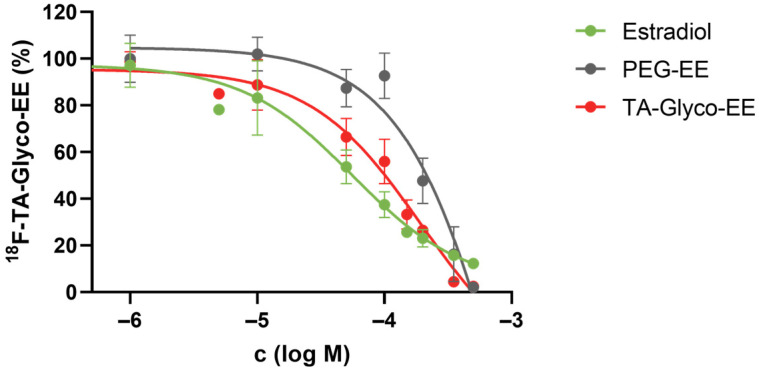
Competitive cellular uptake experiment of ^18^F-TA-Glyco-EE in MCF-7 (ER^+^) breast cancer cells using E2 and EE derivatives. Data are expressed as mean values ± SD from a single experiment performed in triplicate.

Competitive cellular uptake experiments were performed in ER^+^ MCF-7 cells at 37 °C for 60 min, using ^18^F-TA-Glyco-EE as a radioligand and increasing concentrations of E2 or EE derivatives as competitors (Figure 5). The competitive cellular uptake elucidated the findings of the cellular uptake assay. Only at high concentrations of the competitors was the uptake of ^18^F-TA-Glyco-EE completely diminished, which may be due to various transport mechanisms, including passive transport [28]. In the specific assay set up in this work, IC_50_ values of 56 µM (E2), 194 µM (TA-Glyco-EE) and 1274 µM (PEG-EE) were determined for the inhibition of cellular uptake of ^18^F-TA-Glyco-EE in MCF-7 cells (Figure 5).

### 3.3. Biodistribution and Small Animal PET Imaging

**Figure 6:**
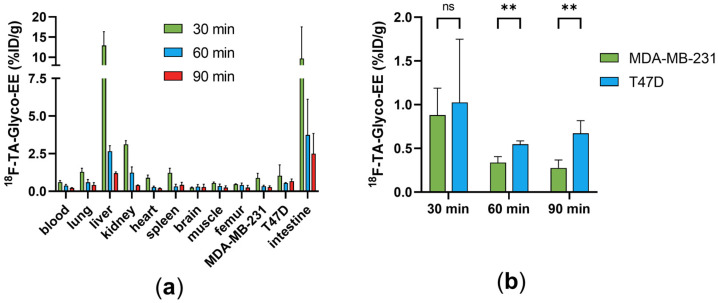
(**a**) Biodistribution of ^18^F-TA-Glyco-EE in T47D (ER^+^) and MDA-MB-231 (ER^−^) tumor-bearing nude mice at 30, 60 and 90 min p.i. Data are expressed in %ID/g as mean values ± SD from three animals per group (*n* = 3–4). (**b**) Statistical comparison of the mean ^18^F-TA-Glyco-EE uptake in ER^+^ and ER^−^ tumors by multiple *t*-test calculations (ns: not significant (*p* > 0.05), ** *p* (60 min) = 0.002, ** *p* (90 min) = 0.003).

The biodistribution of ^18^F-TA-Glyco-EE was determined in T47D, an ER^+^ breast cancer cell line, and MDA-MB-231 (ER^−^) tumor-bearing nude mice (*n* = 3), and the results are depicted in Figure 6. High radioactivity was detected in the liver (13%ID/g) and intestine (10%ID/g) at 30 min p.i., decreasing over 90 min to less than 3%ID/g, revealing the fast hepatobiliary clearance of ^18^F-TA-Glyco-EE. The uptake in blood and all other non-target organs was low for all time points (Figure 6a). The uptake of ^18^F-TA-Glyco-EE in tumors was 1.0%ID/g in T47D tumors and 0.9%ID/g in MDA-MB-231 tumors at 30 min p.i., slightly decreasing over time. The tumor uptake values at 60 min and 90 min p.i. showed statistically significant differences between ER^+^ T47D and ER^−^ MDA-MB-231 tumors (Figure 6b), indicating the ER-specific uptake of ^18^F-TA-Glyco-EE in vivo.

**Figure 7:**
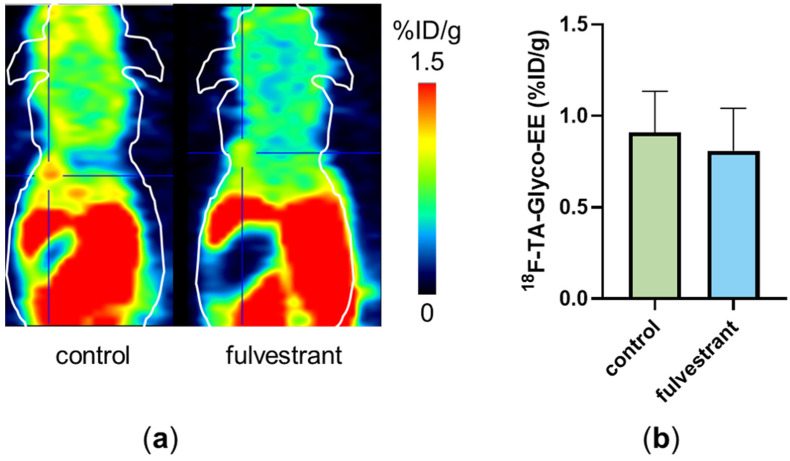
(**a**) Small animal PET scan of a single MCF-7 (ER^+^) tumor-bearing nude mouse injected with ^18^F-TA-Glyco-EE (control, 60 min p.i.). After the first scan, the same mouse received fulvestrant (0.5 mg s.c.), and 48 h later, the ^18^F-TA-Glyco-EE-PET was performed again (“fulvestrant”, 60 min p.i.). The tumor is marked by the crosshair. (**b**) Analysis of tumor uptake of ^18^F-TA-Glyco-EE at 60 min p.i. in MCF-7 (ER^+^) tumor-bearing nude mice (“control”) compared to fulvestrant-pretreated mice (“fulvestrant”). Data are expressed as mean values (in %ID/g) ± standard deviation, each *n* = 4.

Small animal PET imaging was performed with ^18^F-TA-Glyco-EE on ER^+^ tumor-bearing mice over 60 min p.i. (Figure 7). To determine non-specific tumor uptake, fulvestrant (0.5 mg s.c. [29]), as a competitive antagonist, was injected in the same mice and the PET imaging experiment was repeated 48 h later. Pretreatment with fulvestrant resulted in a significantly reduced tumor uptake on PET scans in one of the four animals (Figure 7a). However, in the other three mice, this difference could not be detected (Figure 7b). The low specific uptake of ^18^F-TA-Glyco-EE could be due to the need to implant an E2-releasing pellet in the back of the mice for proper MCF-7 tumor growth, possibly leading to different levels of available free ER or remaining E2 competing with ^18^F-TA-Glyco-EE for ER binding in the tumor.

**Figure 8:**
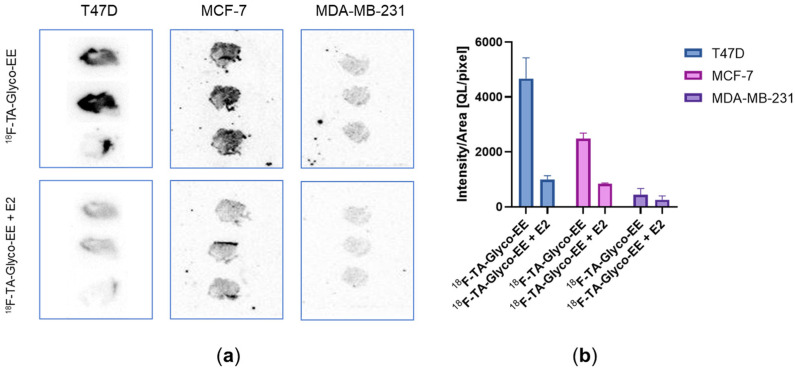
(**a**) In vitro autoradiography of T47D, MCF-7 and MDA-MB-231 tumor slices incubated with either ^18^F-TA-Glyco-EE or ^18^F-TA-Glyco-EE together with E2 (72 µM) for 60 min. (**b**) Analysis of ^18^F-TA-Glyco-EE binding to tumor slices. Data are expressed as mean values of the intensity per area ± SD from 3–4 slices of each group.

Due to the challenging animal model, with ER^+^ xenografts requiring E2-dependent tumor growth, demonstrating specific tumor binding of ^18^F-TA-Glyco-EE in vivo appeared difficult. Therefore, we investigated the specificity of ^18^F-TA-Glyco-EE binding to tumor tissue by the in vitro autoradiography of ER^+^ (MCF-7 and T47D) and ER^−^ (MDA-MB-231) tumor slices (Figure 8). The incubation of the tumor slices for 60 min at room temperature with either ^18^F-TA-Glyco-EE or ^18^F-TA-Glyco-EE together with E2 (72 µM) displayed the high specific binding of ^18^F-TA-Glyco-EE to the ER^+^ MCF-7 and T47D tumor sections and only negligible binding in the ER^−^ MDA-MB-231 tumor sections.

## 4. Discussion

Estradiol derivatives modified in the 17*α*-position address a hydrophobic binding site of ER that tolerates triazolyl substituents [8,19]. This finding, especially the study by Xu et al. dealing with the ^18^F-labeled PEG_2_-triazolyl derivative [^18^F]FETE [8], combined with the previous report on 17*α*-triazolyl EE derivatives bearing various substituents in the 4-position of the triazole ring, e.g., phenyl, p-hydroxyphenyl or alkyl alcohols [19], prompted us to select alternative ^18^F-labeled prosthetic groups with different lipophilicity, including the ^18^F-glycosyl moiety, for the design of new ^18^F-labeled EE derivatives as PET ligand candidates.

Applying a straightforward click chemistry-based strategy, four different ^18^F-labeled EE derivatives were synthesized and evaluated regarding their suitability as radiotracers for ER imaging by PET. All the compounds could be radiolabeled in high yields (AYs between 19 and 44%) in total synthesis times in the range of 27–90 min, which is compatible with the half-life of fluorine-18. Incubation of the radioligands in human serum and plasma showed the high stability of the EE derivatives, except for ^18^F-SiFA-EE, which showed an unfavorable hydrolysis of the Si-^18^F bond. Therefore, ^18^F-SiFA-EE was excluded from further evaluation.

The experimental determination of the logD_7.4_ values for ^18^F-PEG-EE, ^18^F-TA-Glyco-EE and^18^F-Glyco-EE confirmed higher hydrophilicity compared to E2 and FES (logD_7.4_ = 2.5 [30]). This finding supports, on the one hand, our aim to develop alternative tracers with suspected improved liver clearance compared to FES. On the other hand, targeting the nuclear ER requires the passive permeability of the tracer to pass the cell membrane, such that highly hydrophilic properties could be assumed unfavorable. This assumption was confirmed by the in vitro results of ^18^F-Glyco-EE, with the lowest logD_7.4_ of 1.7, which was less suitable for ER targeting as shown by the low cellular uptake in ER^+^ MCF-7 cells. This result is in accordance with the fluoroethyl-triazolyl derivative reported by Tejería et al., which had a similar logD_7.4_ of 1.8 and also showed low cellular uptake in MCF-7 cells [31]. 

On the contrary, ^18^F-PEG-EE could be expected to be a promising tracer for ER targeting, due to its structural similarity to the previously reported [^18^F]FETE, which showed a high tumor uptake of 4%ID/g at 1 h p.i. in MCF-7 tumor-bearing nude mice [8]. In the present study, ^18^F-PEG-EE, with a PEG_3_ chain and logD_7.4_ of 2.2, showed a higher and more specific E2-inhibitable cellular uptake compared to ^18^F-Glyco-EE.

However, the radioligand with the most promising in vitro properties in our series was ^18^F-TA-Glyco-EE, which revealed a moderate lipophilicity (logD_7.4_ = 2.3), as well as a substantially high and ER-specific cellular uptake in MCF-7 cells. The 54-fold increased cellular uptake of ^18^F-TA-Glyco-EE compared to the deacetylated ^18^F-Glyco-EE leads to the conclusion that the absence of polar hydroxyl groups significantly improved the cellular uptake in ER^+^ MCF-7 cells, presumably due to increased passive permeability. The ability of intact ^18^F-TA-Glyco-EE to accumulate in ER^+^ cells was further proven by the HPLC analysis of cell lysates, confirming the relevance of the acetylation of the hydroxyl groups for adequate cellular uptake. In addition, competitive cellular uptake experiments with ^18^F-TA-Glyco-EE showed higher IC_50_ values of PEG-EE compared to the IC_50_ value for self-competition with TA-Glyco-EE, confirming the favorable properties of TA-Glyco-EE for ER-dependent cellular uptake by direct comparison to PEG-EE and Glyco-EE. As expected, we observed deacetylation of ^18^F-TA-Glyco-EE in human serum in vitro, due to carbohydrate esterase activity. Deacetylation of ^18^F-TA-Glyco-EE will also occur in the blood after tracer injection. However, we postulate that at short time points after tracer injection, the acetylation of the tracer and the associated good targeting properties of ^18^F-TA-Glyco-EE may be useful for adequate tumor accumulation. Deacetylation, which occurs in the blood and competes with the uptake of the acetylated form into the tumor, may support the positive effect of rapid liver clearance that has also been observed.

Combined with its fast and high-yielding radiosynthesis (AY = 25% after 77 min), ^18^F-TA-Glyco-EE was therefore assumed to be a promising ^18^F-labeled ER ligand for the PET imaging of ER^+^ breast cancer and was further evaluated in vivo.

The in vivo biodistribution studies and small animal PET imaging of tumor-bearing mice with ^18^F-TA-Glyco-EE faced some challenges. It is necessary to implant an E2-releasing pellet to ensure the proper growth of human MCF-7 and T47D xenografts in mice [32]. However, a remaining significant blood concentration of E2 present in the animals during the in vivo studies would compete with the radiotracer for binding to the ER. 

The biodistribution study was performed in animals with ER^+^ T47D and ER^−^ MDA-MB-231 xenografts. Due to the known liver metabolism of estradiol derivatives, ^18^F-TA-Glyco-EE showed a high uptake in the liver (13%ID/g) at 30 min p.i. However, the radioactivity in the liver substantially decreased over time to reach 1.2%ID/g at 90 min p.i., indicating fast hepatic excretion of the radiometabolites formed from ^18^F-TA-Glyco-EE. We assume that the majority of liver metabolites were formed after deacetylation of ^18^F-TA-Glyco-EE in the blood, resulting in polar metabolites in the liver that were rapidly excreted. The uptake in T47D or MDA-MB-231 tumors was low (<1%ID/g); however, at 60–90 min p.i., a statistically significant ER-specific tumor uptake of ^18^F-TA-Glyco-EE was determined when comparing ER^+^ to ER^−^ tumors. The dynamic small animal PET imaging of ER^+^ MCF-7 xenografts showed the specific tumor uptake of ^18^F-TA-Glyco-EE in only one out of four mice after treatment with fulvestrant to degrade the available estradiol receptors by downregulation [33]. The statistical analysis of all animals did not show a significant difference in tumor uptake of ^18^F-TA-Glyco-EE comparing non-treated to fulvestrant-treated animals. This could be due to remaining high levels of circulating E2 in the mice after E2-dependent tumor growth. It is therefore tempting to speculate that special attention should be paid to testing the E2 level in the animal’s blood prior to the PET scan, to avoid any potential competition with an ER-targeting tracer. In the paper by Xu et al., the sufficient tumor growth of MCF-7 xenografts in vivo without an E2-releasing pellet was reported, resulting in a relatively high tumor uptake of [^18^F]FETE of 4%ID/g (60 min p.i.) that could be significantly diminished by fulvestrant pretreatment to 1.5%ID/g (60 min p.i.) [8]. In contrast, Aliaga et al. [34] compared the tumor uptake of FES in different mouse models, reporting a low uptake (<0.4%ID/g) of FES in MCF-7 and T47D tumors. The authors confirmed a loss of available ER in MCF-7 tumors, suggesting that even after the removal of the necessary E2 depot pellet, the intracellular receptors could still be occupied by E2 for a longer time, resulting in the low uptake of an ER-targeting PET ligand. Our results were consistent with those reported by Aliaga et al., as we observed rather low uptake values of ^18^F-TA-Glyco-EE in MCF-7 tumors of around 1%ID/g.

To verify the presence of estradiol receptor expression in our in vivo model, we performed an in vitro autoradiography using ER^+^ (MCF-7 and T47D) and ER^−^ (MDA-MB-231) tumor slices obtained from the xenografts. The high specific binding of ^18^F-TA-Glyco-EE was observed in both ER^+^ tumor slices, whereas no specific binding was detected in ER^−^ tumor slices. Therefore, our autoradiography study strongly confirmed the presence of ER in T47D and MCF-7 xenografts and revealed a heterogeneous ER distribution in the tumor tissue, while no expression of ER in MDA-MB-231 tumor slices was confirmed. This further corroborates the assumption that the low uptake of ^18^F-TA-Glyco-EE in ER^+^ tumors in vivo may be due to high ER occupation by E2 and not due to the adverse binding properties of ^18^F-TA-Glyco-EE.

## 5. Conclusions

In summary, four different ^18^F-labeled estradiol derivatives with varied lipophilicities were successfully synthesized by a straightforward click chemistry-based strategy using ^18^F-labeled prosthetic groups and evaluated preclinically regarding their ability to specifically bind to ER. The observed hydrolytic instability of ^18^F-SiFA-EE excluded this derivative from further evaluation. ^18^F-PEG-EE, ^18^F-Glyco-EE and^18^F-TA-Glyco-EE showed slight differences in their lipophilicities, but significant differences in their cellular uptake in MCF-7 cells, making ^18^F-TA-Glyco-EE the most promising candidate for in vivo evaluation. Biodistribution, as well as small animal PET studies, revealed a fast clearance from excreting organs such as the liver, most likely due to deacetylation of the tracer in the blood. The lack of a specific uptake of ^18^F-TA-Glyco-EE in ER^+^ tumors in vivo could be explained by ER occupation by E2, since the high specific binding of ^18^F-TA-Glyco-EE was verified by in vitro autoradiography in ER^+^ MCF-7 and T47D tumors in comparison to ER^−^ MDA-MB-231 tumors. In conclusion, ^18^F-TA-Glyco-EE is a promising candidate as an ^18^F-labeled PET tracer for the imaging of ER^+^ breast cancer, but has to be further evaluated in a more suitable animal model before translation into the clinic.

## Figures and Tables

**Table 1:** In vitro data of EE derivatives.

	AY ^1^ (Total Synthesis Time)	logD_7.4_	Plasma Protein Binding	Stability in Human Serum or Plasma
^18^F-PEG-EE	19% (81 min)	2.2 ± 0.1	71 ± 4%	>99% over 2 h ^2^
^18^F-SiFA-EE	44% (27 min)	n.d. ^3,4^	n.d. ^3,4^	<50% after 10 min ^2^
^18^F-TA-Glyco-EE	25% (77 min)	2.3 ± 0.1	79 ± 1%	0% after 30 min ^2,5^
^18^F-Glyco-EE	22% (90 min)	1.7 ± 0.1	n.d. ^3^	>99% over 2 h ^2^

^1^ AY activity yield, defined as non-decay-corrected radioactivity at end of synthesis referred to [^18^F]fluoride. ^2^ values were the same for serum and plasma. ^3^ n.d. not determined. ^4^ due to instability of the compound. ^5^ deacetylation observed in serum and plasma (see Figure 3a).

## Data Availability

All data acquired for this study are stored at the data center of the University Hospital Erlangen, Department of Nuclear Medicine.

## Supplementary Materials

The following supporting information can be downloaded at: https://www.mdpi.com/article/10.3390/cancers16152639/s1, Figure S1: HPLC chromatograms showing co-injection of ^18^F-Glyco-EE (top) and reference compound Glyco-EE (bottom). Figure S2: HPLC chromatograms showing co-injection of ^18^F-TA-Glyco-EE (top) and reference compound TA-Glyco-EE (bottom). Figure S3: HPLC chromatograms showing co-injection of ^18^F-TA-Glyco-EE (top) and reference compound TA-Glyco-EE (bottom). Figure S4: HPLC chromatograms showing co-injection of ^18^F-SiFA-EE (top) and reference compound SiFA-EE (bottom).
