# Supplementary material for: Radiosynthesis and Preclinical Evaluation of 18F-Labeled Estradiol Derivatives with Different Lipophilicity for PET Imaging of Breast Cancer
Source: Cancers (Basel). 2024 Jul 24;16(15):2639. doi: 10.3390/cancers16152639 (PMC11311842; doi:10.3390/cancers16152639)
Supplement: Supplementary file 1 [file cancers-16-02639-s001.zip › cancers-3089822-supplementary.pdf]

Supplementary Material

for

Radiosynthesis and preclinical evaluation of ^{18}F-labeled estradiol derivatives with different lipophilicity for PET imaging of breast cancer

Anna Friedel [1], Olaf Prante [1,2] and Simone Maschauer [1,*]

[1]Friedrich-Alexander University Erlangen-Nürnberg (FAU), Department of Nuclear Medicine, Molecular Imaging and Radiochemistry, 91054 Erlangen, Germany

[2]FAU NeW – Research Center New Bioactive Compounds, Friedrich-Alexander-Universität Erlangen-Nürnberg, 91058 Erlangen, Germany

*****Correspondence: simone.maschauer@uk-erlangen.de

Table of Content:

Figure S1-S4: Coinjections of ^{18}F-labeled EE derivatives and their corresponding reference compounds

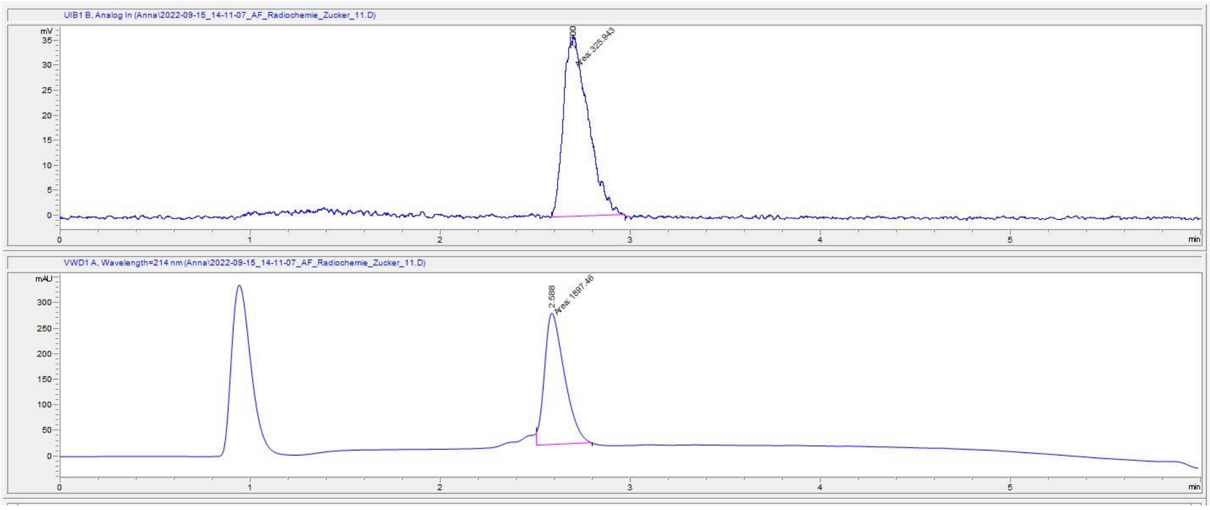

Figure S1: HPLC chromatograms showing coinjection of ^{18}**F-Glyco-EE** (top) and reference compound **Glyco-EE** (bottom).

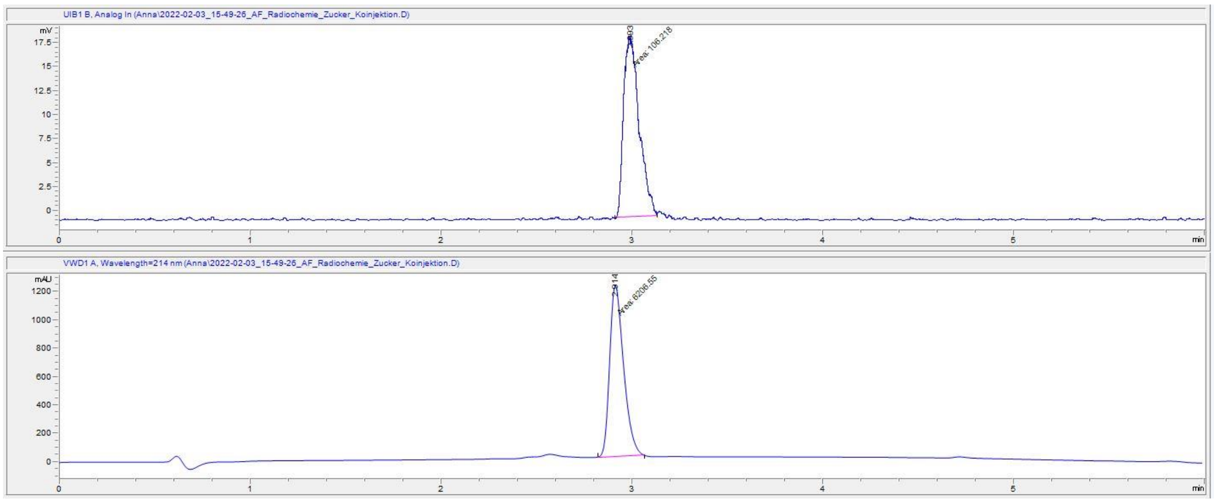

Figure S2: HPLC chromatograms showing coinjection of ^{18}**F-TA-Glyco-EE** (top) and reference compound **TA-Glyco-EE** (bottom).

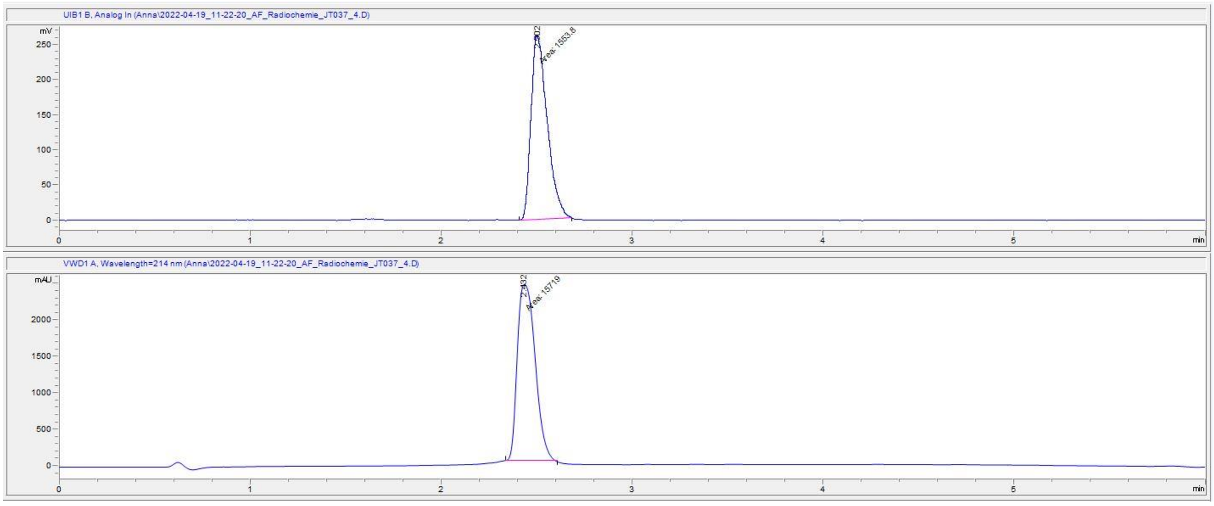

Figure S2: HPLC chromatograms showing coinjection of ^{18}**F-TA-Glyco-EE** (top) and reference compound **TA-Glyco-EE** (bottom).

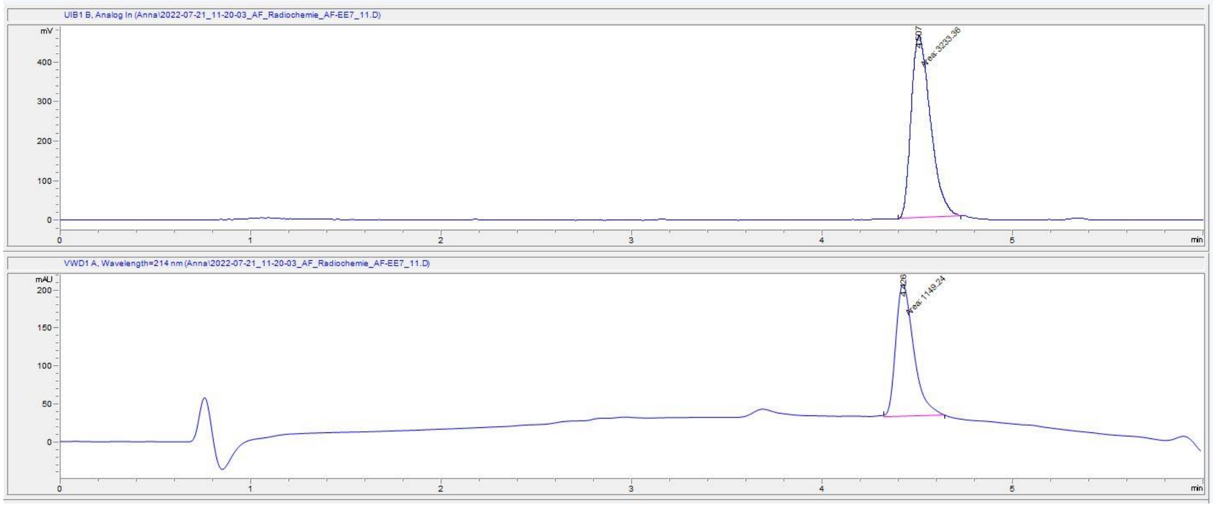

Figure S4: HPLC chromatograms showing coinjection of ^{18}**F-SiFA-EE** (top) and reference compound **SiFA-EE** (bottom).
